# Hydroxyapatite in Oral Biofilm Management

**DOI:** 10.1055/s-0039-1695657

**Published:** 2019-10-01

**Authors:** Frederic Meyer, Joachim Enax

**Affiliations:** 1 Research Department, Dr. Kurt Wolff GmbH and Co. KG, Bielefeld, Germany

**Keywords:** biofilm, caries, hydroxyapatite, periodontitis

## Abstract

Particulate hydroxyapatite, Ca _5_ (PO _4_ ) _3_ (OH), shows a good biocompatibility and is used as a biomimetic ingredient in dental care formulations due to its similarity to human enamel. Numerous studies show its efficiency, for example, in reducing dentin hypersensitivity, and in the remineralization of enamel and dentin. In addition, oral care products with hydroxyapatite improve periodontal health under *in vivo* conditions. This review article summarizes data on the effects of hydroxyapatite particles in oral biofilm management. Two databases (PubMed and SciFinder) were searched for studies using specific search terms. In contrast to frequently used antibacterial agents for biofilm control, such as chlorhexidine, stannous salts, and quaternary ammonium salts, hydroxyapatite particles in oral care products lead to a reduction in bacterial attachment to enamel surfaces in situ without having pronounced antibacterial effects or showing unwanted side effects such as tooth discoloration. Furthermore, antibacterial agents might lead to dysbiosis of the oral ecology, which was recently discussed regarding pros and cons. Remarkably, the antiadherent properties of hydroxyapatite particles are comparable to those of the gold standard in the field of oral care biofilm management, chlorhexidine *in situ* . Although biomimetic strategies have been less well analyzed compared with commonly used antibacterial agents in oral biofilm control, hydroxyapatite particles are a promising biomimetic alternative or supplement for oral biofilm management.

## Introduction

Bacterial biofilms are complex structures and mostly consist of several species that are embedded in a matrix of extracellular polymeric substances. 1 2 3 4 5 The presence, growth, and metabolism of oral biofilms are the main causes for dental caries and periodontitis. 4 5 6 These two diseases affect more than 2.44 billion people (active caries with permanent dentition) 7 and 743 million people (severe periodontitis) worldwide. 8

Therefore, a main preventive measure in oral care is to control oral bacterial biofilms. 4 This can be mainly achieved by mechanical plaque removal, for example, tooth brushing (manual or electric toothbrush), flossing, and others, as well as by the toothpaste formulation (i.e., abrasives). 9 10 11 12 13 The mechanical biofilm removal can be supported by antibacterial agents in toothpastes or mouthwashes. 14 Frequently used antibacterial agents are, for example, chlorhexidine, metal salts, quaternary ammonium salts, and others. 4 15 16 17 18 However, daily use of products with some of these antimicrobials might lead to unwanted side effects; for example, chlorhexidine and stannous salts lead to extrinsic stain of teeth. 15 19 20 Consequently, dental research is focused on new approaches in oral biofilm management. 21 22 23 24 25 Hence, biomimetic approaches are promising, because they mimic structures or processes that have been evolutionarily optimized by nature over a long period of time. 26 27 28 In the field of enamel-inspired materials, hydroxyapatite, Ca _5_ (PO _4_ ) _3_ (OH), as a biomimetic oral care ingredient, has gained increasing attention in the last decades. 24 26 27 29 30 31 32 33 34 35 Hydroxyapatite shows a good biocompatibility and has been widely used for biomedical applications such as bone cements and implant coatings for many years. 36 37 38 Regarding preventive dentistry, products with hydroxyapatite offer a broad range of applications, that is, prevention of dental caries, prevention of gingivitis/periodontitis, and dentin hypersensitivity. 29 33 34 39 40 41 42 43 44 45 Interestingly, in situ studies show remarkable effects of hydroxyapatite particles, reducing initial bacterial colonization to enamel and oral surfaces. 24 35 46 ,

This review summarizes published studies on hydroxyapatite with respect to biofilm management and presents possibilities for further research avenues.

## Study Selection

*In vivo, in situ,* and *in vitro* studies on hydroxyapatite in oral care, recently reviewed by Enax and Epple 26 and Meyer et al, 27 were included into this review. In addition, two databases (PubMed and SciFinder) were used for literature search with following search terms: “Hydroxyapatite” AND (“ *in vitro* study” OR “ *in situ* study” OR “ *in vivo* study” OR “clinical study” AND “remineralization” OR “caries” OR “bacteria” OR “plaque” OR “biofilm” OR “periodontitis”) OR “oral care.” The references were screened for relevance and included in this review, respectively.

## Concepts in Modern Biofilm Management

### Classical Antibacterial Agents

Antibacterial agents, frequently used in oral care products such as toothpastes and mouthwashes, are summarized in Table 1 . Regarding biofilm control/plaque reduction, the well-known gold standard is chlorhexidine. Chlorhexidine shows a wide effect of spectrum against Gram-positive and Gram-negative bacteria and is known to show a high substantivity. However, long-term use of chlorhexidine increases the risk of side effects such as taste alteration (dysgeusia) 47 or staining of the tooth surface. 48 The antibacterial potential of other substances, for example, metal salts, quaternary ammonium salts, and natural extracts, is lower compared with chlorhexidine, 21 but can be used on a daily basis and is commonly introduced in oral care products. It is important to know that the antibacterial effect of metal salts (e.g., zinc chloride and stannous fluoride) is mainly attributed to the metal ion (i.e., Zn ^2+^ and Sn ^2+^ ) and not to its counterion. 15 This is also true for amine fluorides where the cationic surfactant (ammonium salt) shows the antibacterial effect, and not the fluoride ion itself. 10 11 49 Furthermore, zinc ions are not only known for their antibacterial effect but also to prevent oral malodor by inhibiting volatile sulfur compounds. 50 51 52 Regarding antimicrobial effects, zinc ions show a high substantivity in the oral cavity. 53 Zinc ions can inhibit bacterial metabolism (i.e., glycolysis and trypsin-like protease), leading to a reduced biofilm formation. 15 Compared with other antibacterial metal salts (i.e., Sn ), zinc does not stain the enamel surface. 15

**Table 1:** Examples of biofilm controlling agents used in oral care products such as toothpastes and mouthwashes (in alphabetic order)

Substance classes	Examples
Amine fluorides	Olaflur, dectaflur (antibacterial effect is based on the cationic amine)
Bisbiguanides	Chlorhexidine
Calcium phosphates	Hydroxyapatite (biomimetic approach; reduction of bacterial colonization without antibacterial effects)
Phenols	Triclosan
Quaternary ammonium salts	Cetylpyridinium chloride (antibacterial effect is based on the cationic amine)
Stannous salts	Stannous chloride, stannous fluoride (antibacterial effect based on Sn ^2+^ -ions)
Surfactants	Sodium lauryl sulfate, sodium cocoamphoacetate
Zinc salts	Zinc chloride, zinc citrate, zinc PCA (antibacterial effect is based on Zn ^2+^ -ions)
Abbreviation: PCA, pyrrolidone carboxylic acid.	

Nevertheless, all the above-mentioned agents have common property that they can kill both harmful and beneficial bacteria. However, the overall goal in biofilm management is to keep the oral microbiome in a homeostatic state. 5 54 This means antimicrobials might lead to a selection of potentially pathogenic bacteria and consequently to a dysbiosis of the microbiome. 5 54

Therefore, research also focuses on alternative concepts, that is, on biomimetic approaches keeping the microbiome in balance. 4 10 11 23 24

### Particulate Hydroxyapatite in Biofilm Management

Different types of hydroxyapatite are used in oral care formulations worldwide (e.g., hydroxyapatite and zinc hydroxyapatite). 26 Numerous studies on hydroxyapatite in dental care have been published in the last years. 26 27 30 For example, a (fluoride-free) zinc hydroxyapatite-containing toothpaste showed a comparable clinical performance in periodontitis patients compared with an antibacterial fluoridated toothpaste with amine fluoride (Olaflur) and stannous fluoride, for example, in reduction of bleeding on probing. 33 In addition, a zinc hydroxyapatite mouthwash showed a reduction in plaque accumulation and gingivitis in children in vivo. 42

Details on biofilm management using hydroxyapatite have been thoroughly analyzed in several in situ 24 35 and *in vivo* studies. 46

These studies analyzed the influence of a hydroxyapatite mouthwash as well as hydroxyapatite particles and the liquid phase of the mouthwash. Antibacterial effects could be mainly assigned to the liquid phase, whereas hydroxyapatite particles show antiadherent properties, that is, reduction in initial bacterial colonization to the enamel surface. 35 Interestingly, pure hydroxyapatite particles dispersed in water (i.e., without any apatite substituents such as zinc or other additives commonly used in oral care products) reduced the bacterial attachment to enamel surfaces comparable to the gold standard chlorhexidine in situ, without any antibacterial effect. 24 Studying the raw material is very important to analyze its efficiency and efficacy, because other ingredients such as ethanol (usually used in combination with essential oils), 55 surfactants, preservatives, and others as well as an acidic pH value (e.g., formulations with amine fluoride) in oral care formulations may have an influence on the results of biofilm management. 4 Besides in situ studies, in vitro studies using subminimal inhibitory concentration show inhibitory effects of zinc hydroxyapatite products against cariogenic biofilms. 56 57 This means biofilm formation is inhibited, but bacteria are not killed. In addition, it has been reported that the use of a mouthwash, containing zinc hydroxyapatite and zinc L-pyrrolidone carboxylate, leads to a reduced bacterial attachment to suture threads. 46 In summary, these findings make hydroxyapatite a potent biomimetic alternative to classical antibacterial agents for oral care use. In addition to the existing studies, future research needs to focus on the incorporation of hydroxyapatite particles into oral biofilms and thus a clarification of the mode of action. 58 Synergistic effects in biomimetic oral biofilm management may be achieved by combining hydroxyapatite particles with saliva proteins, such as lactoferrin or other enzymes. 23 59

## Conclusions

Due to its high biocompatibility as well as its structural and chemical similarity to human enamel, hydroxyapatite is a promising oral care ingredient. Studies show that it reduces the bacterial attachment to enamel surfaces similar to chlorhexidine in situ, but without killing the bacteria. In addition, hydroxyapatite particles offer other beneficial effects in the oral cavity, for instance, remineralization of enamel and dentin as well as prevention of dentin hypersensitivity. Although more research is needed to understand hydroxyapatite’s mode of action in reducing the bacterial attachment to the enamel surface and to compare its efficiency toward other antibacterial substances in vivo, hydroxyapatite may be a promising biomimetic alternative or supplement for oral biofilm management.
